# Higher-order brain processes, rather than early processing, underlie sensory problems in ME/CFS: evidence from ERPs

**DOI:** 10.3389/fmed.2026.1842841

**Published:** 2026-06-18

**Authors:** Sanjay Kumar, Alfred Veldhuis, Farzaneh Yazdani

**Affiliations:** 1 Department of Psychology, Social Work and Public Health, Oxford Brookes University, Oxford, United Kingdom; 2 School of Life Sciences, University of Roehampton, London, United Kingdom; 3 Occupational Therapy, Oxford Brookes University, Oxford, United Kingdom

**Keywords:** brain, chronic sensation, cognition, electroencephalography, fatigue syndrome

## Abstract

**Introduction:**

Patients with Myalgic Encephalomyelitis (ME)/Chronic Fatigue Syndrome (CFS) experience significant sensory problems that affect their personal, social and occupational life. However, there is no clear understanding of how the sensory problems manifest in ME/CFS. Neuroimaging studies have provided indirect evidence of the involvement of sensory brain areas in ME/CFS. This novel systematically examined the role of early sensory processing and late information processing brain systems in ME/CFS patients.

**Methods:**

The participants consisted of 31 ME/CFS patients and 30 healthy matched controls. Measures of subjective experience of sensory problems as well as event-related brain potentials (ERPs) on an auditory paired click task and an auditory oddball task were collected.

**Results:**

ME/CFS patients reported significantly higher sensory problems compared to the control group. On the ERP measures, the ME/CFS group was not significantly different on the P50 suppression index than the control group. However, the ME/CFS group showed a significantly reduced P300 potential compared with the control group.

**Discussion:**

These findings suggest that the higher-order control-based brain mechanism contributes to the sensory problems experienced by ME/CFS patients. These findings could have profound implications for targeted interventions directed towards higher-order brain systems, rather than the sensory systems, to address challenges related to sensory processing problems in ME/CFS.

## Introduction

Chronic Fatigue Syndrome (CFS) or Myalgic Encephalomyelitis (ME) is a neurological disorder affecting about 0.76% of the population, with a higher female-to-male ratio (1). ME/CFS is characterized by debilitating fatigue lasting at least 6 months, worsening with physical and mental activity but not fully improving with rest. Other symptoms include neurological impairments affecting sensory and cognitive functions, and sleep issues (2). This suggests the brain plays a critical role in the disease process of ME/CFS (3). Despite advances in understanding its etiology and pathophysiology, the mechanisms of sensory and cognitive impairments in ME/CFS remain poorly understood. Previous research on the brain mechanisms involved in the cognitive problems associated with ME/CFS, including decreased brain activity, gray and white matter changes, altered brain activation patterns, and the recruitment of additional brain areas, all linked to cognitive performance (4, 5). In contrast, sensory processing in ME/CFS patients has not been extensively investigated.

In a constantly changing environment with many competing stimuli, ME/CFS patients often feel overwhelmed, which impacts their personal, social, and professional lives and affects their mental and physical health. Sensory impairments could be critical for guiding treatment planning, setting treatment goals, and adjusting the workplace or school setting. Recent guidelines are considering the recognition of sensory symptoms for diagnostic purposes (6). However, it is not currently a part of the diagnostic criteria of many diagnostic systems, even though ME/CFS patients commonly report sensory problems (7). A recent study has shown that a significantly higher percentage of ME/CFS patients reported hypersensitivity to light and noise than in another chronic condition, multiple sclerosis, indicating that the chronicity of the condition does not explain the hypersensitivity observed in ME/CFS (8). It was further observed that hypersensitivity was associated with more reported symptoms. Hypervigilance and sympathetic activities have been reported in ME/CFS patients (9). Hypervigilance leads to increased stimulus intake, which the brain does not adequately process due to reduced cerebral blood flow. Increased stimulus uptake and concomitantly reduced brain processing capacity may lead to stimulus overload, which results in hypersensitivities against sensory stimuli such as light, noise, and smells (10). It has also been suggested that hypersensitivity to light and sound is unique in ME/CFS and rarely seen in other fatigue-related conditions (11). Hence, it warrants a deeper understanding of sensory processing abnormalities in ME/CFS.

Sensory systems are hierarchically organized, allowing stimuli, like auditory information, to ascend through brain regions to the primary sensory cortex (auditory cortex). From there, stimuli project to secondary sensory cortices that connect to areas beyond the sensory pathway, like the prefrontal cortex (PFC). The PFC modulates sensory processing through feedback projections (12). Early sensory processing appears stimulus-driven and reliant on the integrity of sensory systems, whereas later processing seems dependent on top-down influences. The top down and bottom up systems can interact dynamically to allow effective stimulus processing (13–15). A key question is how sensory processing is impacted in ME/CFS patients, affecting either early perceptual or later response-related processes.

Altered brain activities in the sensory brain regions has been observed in ME/CFS. For example, a functional magnetic resonance imaging (fMRI) study found reduced, task-independent responsiveness of the auditory cortices during the fatigue-inducing period in ME/CFS patients, which was not observed in healthy control participants (16). Furthermore, they observed an association between the reduction of auditory cortices' responsiveness and reported fatigue in these individuals, suggesting a possible role of the sensory brain areas in mental fatigue observed in ME/CFS. Reduced function brain connectivity of the salience network was proposed as a mechanism associated with problems in detecting and integrating salient sensory information in ME/CFS (17). Cook et al. also found that activity in the parietal region (a multi-sensory integration area) was positively associated with self-reported fatigue and difficulty concentrating, suggesting the involvement of sensory processing systems in ME/CFS.

Sensory processing is a fast process that can be accurately investigated with electroencephalogram (EEG) techniques, as EEG has excellent temporal resolution to understand brain- related processes at different time points. Early components reflect sensory processing and categorization, while later ERP components, like N200 and P300, relate to attentional and executive control processes (18). However, few studies have examined sensory processing in ME/CFS using EEG methods. EEG signal-based coherence analysis has been shown to distinguish ME/CFS patients from healthy controls and depressed participants with excellent accuracy (19). The distinguishing EEG factors were more prominent in the temporal region, a putative site for auditory signal processing, amongst other functions. However, some studies found no clear indication of sensory processing being impacted in people with ME/CFS in the EEG literature. For example, Prasher et al. (20) found no differences in event-related potentials (ERPs) for sensory components measuring brainstem potentials via a polarity click simulation, visual potentials, or somatosensory potentials. In contrast, the authors did find clear differences in the cognitive ERPs as the P3 was significantly delayed or absent in 52% of their participants on an auditory discrimination task. Contrarily, Polich et al. (21) found no ERP (N1, P2, N2, P3) component differences between individuals with ME/CFS and controls on an auditory oddball task. This lack of consistent support in the EEG literature on sensory processing in ME/CFS conflicts with other neurophysiological and behavioral findings. Sensory processing dysfunctions are observed in various neurological disorders like traumatic brain injury (22), migraine (23), and tinnitus (24).

As sensory processing is a fast, multi-stage, both bottom-up and top-down mediated process, the current study aims to address sensory processing problems in ME/CFS using the excellent temporal resolution of ERP. In this research, we asked if the early bottom-up sensory gating, which prevents information overload by suppressing trivial and repetitive sensory information (P50, N100) (25), and/or later cognitive control top-down processes (N200, P300) that regulate sensory processing, may be affected in ME/CFS. Understanding early and late sensory processing problems in ME/CFS through associated ERP components will inform potential pharmacological intervention or cognitive remediation pathways.

## Material and methods

### Participants

A total of 65 participants provided written informed consent to the study. Four were removed due to technical issues, leaving 61 participants divided into two groups: Individuals with ME/CFS; and a control group of age-matched individuals without ME/CFS or neurological conditions. The ME/CFS group's age ranged from 19 to 76 years (M = 45.87, SD = 15.36), with 29 females and two males. The control group's age ranged from 21 to 74 years (M = 44.70, SD = 16.37), with 16 females and 14 males. There was no significant difference of age between groups, *t*_(59)_ = 0.29, *p* = 0.774. There was, however, a significant difference of gender, χ^2^(1, *N* = 61) = 12.74, *p* < 0.001, with more women in the ME/CFS group (93.50%), compared to the control group (53.30%). All participants in the ME/CFS group had a diagnosis of ME, predominantly made by specialists (23) rather than GPs (8). See Table 1 for an overview of the sample characteristics.

**Table 1:** Descriptive statistics table.

Demographics	Frequency (%), *Means (SD)*			
	Control group		ME/CFS group	
Sample size	30		31	
Age	44.70	(SD 16.37)	45.87	(SD 15.32)
Gender	
Male	14	(46.70)	2	(6.50)
Female	16	(53.30)	29	(93.50)
Ethnicity	
White	27	(90.00)	31	(100.00)
Other	3	(10.00)	0	(0.00)
Diagnosis	
ME/CFS	0	(0.00)	32	(100)
Anxiety	3	(10.00)	17	(54.80)
Depression	3	(10.00)	4	(12.90)
Fibromyalgia	0	(0.00)	8	(25.80)
Allergies	4	(13.30)	13	(41.90)
Other	5	(16.70)	12	(38.70)
HADS	
Anxiety	4.67	(SD 3.99)	7.71	(SD 4.15)
Depression	4.80	(SD 2.04)	7.71	(SD 3.53)
DSQ-SF	
Severity	5.67	(SD 5.54)	27.48	(SD 8.05)
Frequency	5.90	(SD 5.45)	34.19	(SD 9.34)
SP2	
Low registration	27.97	(SD 8.11)	42.61	(SD 7.06)
Sensation seeking	44.67	(SD 7.22)	37.71	(SD 8.73)
Sensory sensitivity	34.27	(SD 9.80)	49.13	(SD 9.05)
Sensation avoiding	35.70	(SD 10.67)	48.55	(SD 9.29)

DSQ-SF, DePaul Symptom Questionnaire –Short Form; HADS, Hospital Anxiety and Depression Scale; ME/CFS, Myalgic Encephalomyelitis/Chronic Fatigue Syndrome; SP2, Sensory Profile Questionnaire.

The Oxford Brookes University Research Ethics Committee (UREC: 191321) approved the study, and the research adhered to all guidelines and regulations involving experiments with human participants. All participants received £30 for participation, plus up to £30 for travel expenses.

### Electroencephalogram (EEG) data collection

All EEG experiments were conducted on a PC desktop with a 24” monitor at a 100 Hz refresh rate, positioned 100 cm from the chair to the monitor. Behavioral experiments used E-Prime 3. EEG was continuously recorded via a 64-channel elastic cap based on the 10/20 layout, using the BioSemi ActiveTwo system (26). Four additional flat electrodes were placed: one on each mastoid and one 1 cm from both canthi to record the Electrooculogram (eog). To ensure high signal quality, all channels were checked for stable offsets, and each active electrode was checked for rapid oscillations, ensuring the signal remained between ±25 mV. A 512 Hz sampling rate was used, and all data were re-referenced offline using the average of the two mastoids. The data were filtered with a band-pass of 0.1 Hz to 25 Hz, plus a 50 Hz notch filter. Independent component analysis (ICA) identified ocular artifacts. For each Event-Related Potential (ERP) of interest, a specific epoch was created with a 200 ms baseline. Continuous EEG data were epoched from −200 to 600 ms. Channels within an epoch containing voltage steps over 50.0 mV, voltage differences over 300.0 mV within trials, or differences less than 0.50mV within 100 ms intervals were highlighted automatically, followed by a manual acceptance or rejection of the epochs. About 1% of the trials were rejected at this stage. After artifact rejection, the epochs were averaged for each participant. Brain Vision Analyser 2 (27) was used for offline EEG processing.

### Material

#### Questionnaires

The self-assessed questionnaires included three sets of questions via Qualtrics software in a fixed order, covering basic demographics: ME/CFS diagnosis confirmation, diagnosis year, illness duration, medical history, and two additional questionnaires described below, plus one Questionnaire presented via the presented using the Pearson's online Portal Q-Global (28).

#### Hospital anxiety and depression scale (HADS)

The HADS (29) is a 14-item scale (7 for depression and 7 for anxiety) assessing depression and anxiety in individuals with physical illnesses. Higher scores on the subscales indicate greater levels of these conditions.

#### DePaul symptom questionnaire –short form (DSQ-SF)

The DSQ-SF (30) is a 14-item self-report questionnaire assessing the frequency and severity of symptoms across 8 domains: Fatigue, Post Exertional Malaise, Sleep, Pain, Neurocognitive, Autonomic, Neuroendocrine, and Immune, on a 0–4 scale. Higher scores indicate more severe symptoms.

#### Sensory profile questionnaire (SP2)

A final questionnaire was the SP2 (31) which is a 60-item self-reported tool that evaluates sensory experiences across six domains: taste/smell, movement, vision, touch, activity, and audition. Participants' responses categorize these experiences into four quadrants: Low Registration (high threshold, passive response), Sensation Seeking (high threshold, active response), Sensory Sensitivity (low threshold, passive response), and Sensation Avoiding (low threshold, active response).

#### Experiments

##### Auditory oddball task

A standard auditory oddball used two pure tones: 600 Hz and 1,600 Hz (60 db), with 600 Hz being more frequent (80% of trials) than 1,600 Hz (20%). Each tone lasted 60 ms with a 10 ms rise and fall. A total of 300 tones were randomly, with an intertrial interval of 500 ms to 700 ms. Participants pressed the spacebar only for infrequent tones, while a white fixation cross was displayed on a gray background, instructing them to focus on it.

##### Auditory paired-click task

The auditory paired-click paradigm involved identical clicks (20 ms, 2,000 Hz) with 500 ms between each (S1 and S2). The intertrial interval varied from 4,500 ms to 9,500 ms. A total of 60 pairs were presented, with a white fixation cross on a gray background throughout the experiment. Participants were asked to focus on the fixation cross, and no response was required.

### Procedure

**Figure 1:**
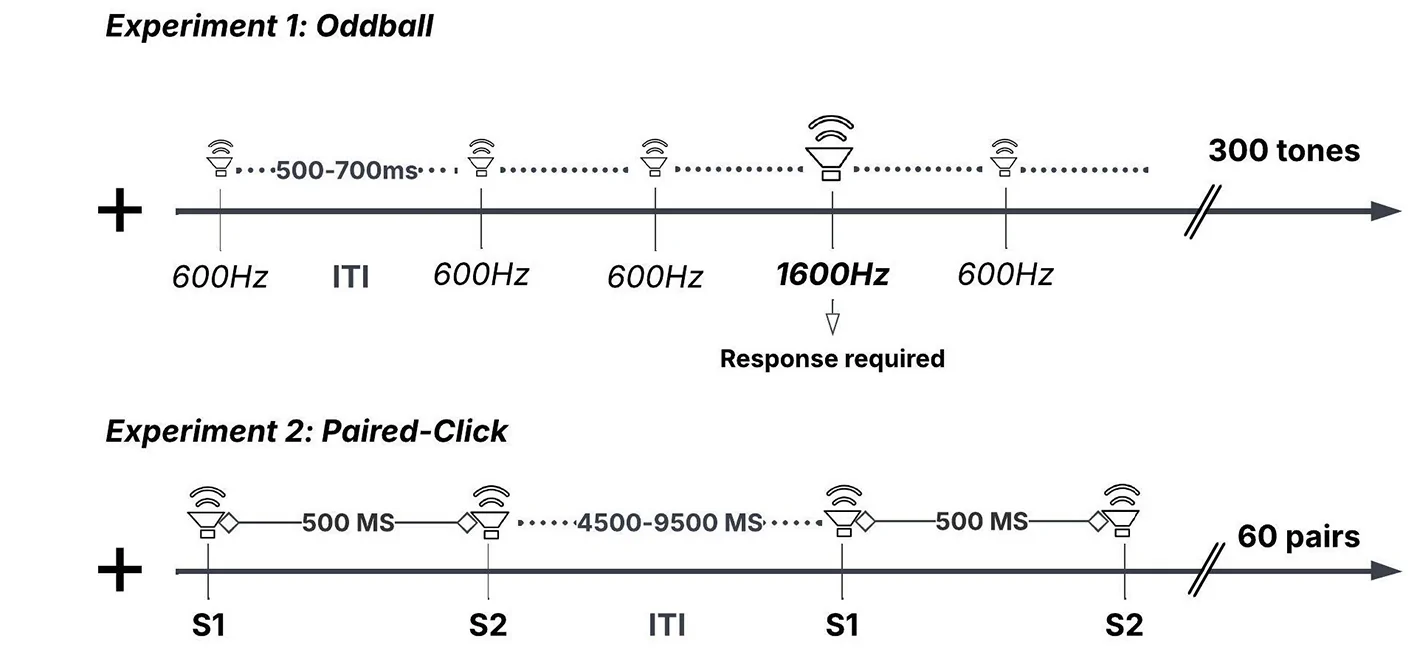
Schematic representation of Experiment 1: Oddball **(Top)** and Experiment 2: Paired-Click **(Bottom)**. Experiment 1 had low pitch (600Hz) and high pitch (1,600Hz), frequent and infrequent stimuli. The high-pitched stimuli occurred randomly in the sequence. For the Paired-Click, all stimuli were 2,000Hz, and S1 and S2 were always 500ms apart.

A week before their study participation, participants received an email reminder with a link to the questionnaires, which needed to be completed before arrival. Completing the questionnaires took about 20 to 30 min, allowing participants to do so at home at their own pace. They later visited the EEG lab at Oxford Brookes University to sign a consent form, separate from the questionnaires' consent process. Participants were seated in a dimly lit, sound-attenuated room with a keyboard about 100 cm from the monitor. The EEG procedure was explained, and they read instructions on the screen. The experiment order was set: first the Oddball task, then the Paired-Click paradigm. The Oddball task began with a 10-trial training phase to familiarize participants. They were instructed to press the spacebar upon hearing a high-pitched tone (1,600 Hz). Midway, the task paused for a self-paced break before proceeding. The paired click task followed, where participants focused on a fixation cross while paired tones were presented. Overall, the EEG experiment lasted about 30 min. Please see Figure 1 for an overview.

### Analysis

The main EEG analyses focused on comparing the means of ERPs scores against the “Group” binary variable consisting of the participants in the control group vs. the ME/CFS participants using a 2 × 2 Mixed ANOVA. As Gender and the HADS scores (see results section) significantly differed between control and ME/CFS groups, and considering their potential impact on ERPs components (32–34), gender, HADS-A and HADS-D were controlled for in the analysis. Bonferroni corrections were applied to adjust for multiple comparisons. The assumptions of normality and equal variance (Levene's test) were met for all ANOVAs. The assumption of normality of the residuals was inspected using Q-Q plots, and the N100 S1 and S2 for the control group were not normally distributed. Similarly, the S1 for the P50 was not normally distributed. Nonetheless, the factorial ANOVA is considered to be robust against moderate violation of normality. All other conditions were normally distributed.

For the Oddball experiment, the mean ERPs for the High-frequency vs. Low-frequency (i.e., oddball) stimuli were compared against the grouping variable. The P300 ERP is a more parietal component and was measured by averaging waveforms for each participant and pooling the electrodes CPz, P1, P2, POz, and Pz, capturing the midline electrodes and reflecting the centro-parietal distribution of the P300, with a maximal amplitude often reported at Parietal electrodes (35, 36). Therefore, a grouping around the Pz electrode was chosen. Previous research has used a similar electrode grouping (37). The selection of the P300 peak was based on the average P300 peak observed in the grand average waveform, which occurred around 360 ms after stimulus onset, with mean amplitude calculated over a 50 ms period around the peak (335 to 385 ms).

For the Paired click experiment the ERPs scores of S1 vs. S2 was compared against Group. The same analysis was applied for the P50, N100, and P200 ERP components. The detection of P50 peaks on the Cz electrode for each participant began with semiautomatic detection of N100 between 85 and 150 ms. If no clear N100 was detected, a 0–300 ms window was used to identify the most negative peak. P50 was identified as the most positive peak preceding N100 in the 35–85 ms window; otherwise, it was the most pronounced peak before N100. P200 is the most positive peak following N100, but before 300 ms after stimulus onset. For S2, the P50 of S1 must be within 10 ms of 2′s P50, while for N100/P200, the limits are 40 and 80 ms, respectively. If no peaks are observable, the component is fully attenuated, unless visual inspection shows these components (with a clear central or fronto-central scalp distribution) outside the specified time windows (38). Although this procedure was strictly followed, the nature of semi-automatic peak detection does allow for the potential of experimenter bias.

The three questionnaires were all compared against the grouping variable using independent *t*-tests. The assumption of normality was assessed using Q-Q plots; although the ME/CFS group was normally distributed, the control groups showed moderate deviation from normality for the DSQ-SF and the HADS. Given a sufficiently large sample size, the independent *t*-test is reported; however, the results were also assessed using a Mann-Whitney U-test, and the same pattern of findings persisted. The scores were also correlated with the ERP outcomes, using Pearson's coefficient.

## Results

### Questionnaires

#### DSQ-SF

**Figure 2:**
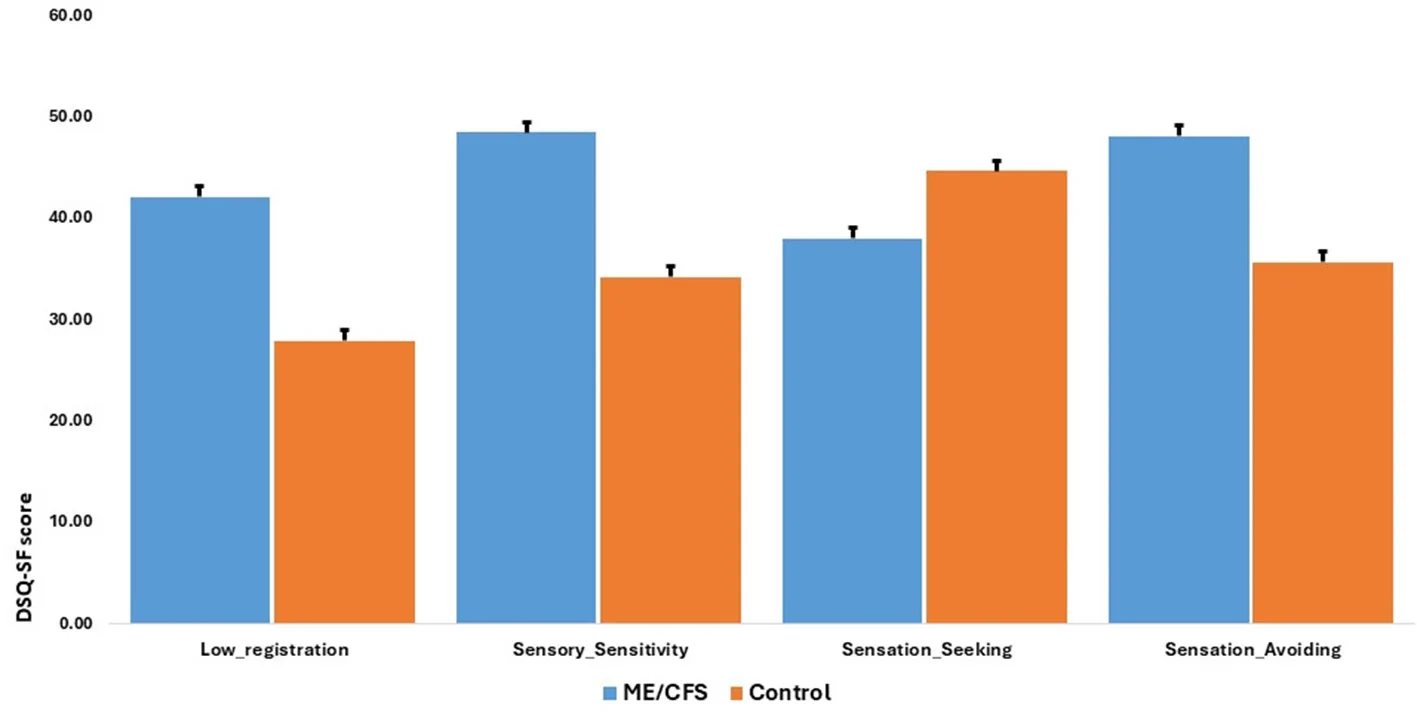
Mean scores on the Sensory Profile questionnaire for the ME/CFS and Control groups. The error bar represents 1 standard error of the mean. All comparisons between ME/CFS and Control are significant.

The ME/CFS group had significantly higher fatigue severity (M = 27.48, SD = 8.05) and frequency score (M = 34.19, SD = 9.34) when compared to the control group (severity M = 5.67, SD = 5.54; frequency M = 5.90, SD = 5.45), both showing a large effect size: DSQ-S: t_(53.30)_ = 12.36, p <0.001, 95%, d = 3.15, CI [−25.36, −18.28]; DSQ-F: t_(48.60) = _ 14.51, p <0.001, d = 3.69, 95% CI [−32.21, 24.38] Figure 2.

#### HADS

The anxiety and depression scales both revealed a significant difference between control and ME/CFS. HADS-A, *t*_(59)_ = 2.92, *p* = 0.005, *d* = 0.75. 95% CI [−5.13, −0.96], revealed medium effect size with control scoring lower on anxiety (M = 4.67, SD = 3.99) compared to ME/CFS (M = 7.71, SD = 4.15); and HADS-D, *t*_(48.32)_ = 3.95, *p* < 0.001, *d* = 1.00, 95% CI [−4.39, −1.43] showing a large effect size with the control group scoring lower on depression (M = 4.80, SD = 2.04) than ME/CFS (M = 7.71, SD = 3.53).

#### Sensory profile

Compared to the control group, participants in the ME/CFS scored higher on: Low Registration, *t*_(59)_ = 7.53, *p* < 0.001, *d* = 1.93, 95% CI [−18.54, −10.75]; Sensory Sensitivity, *t*_(59)_ = 6.16, *p* < 0.001, *d* = 1.58, 95% CI [−19.69, −10.03]; and Sensory Avoiding, *t*_(59)_ = 5.02, *p* < 0.001, *d* = 1.29, 95 % CI [−17.97, −7.73], all with a large effect size. Last for Sensation Seeking ME/CFS participants scored lower compared to the control group, *t*_(59)_ = 3.39, *p* = 0.001, *d* = 0.87, 95%CI [2.85, 11.07], showing a large effect size as well. See Figure 2, and Table 1 for the means and SDs.

### ERP results

#### Oddball paradigm

A significant interaction, with a medium effect size, was revealed: *F*_(1, 56)_ = 6.33, *p* = 0.015, η*p*^2^ = 0.10. For the frequent trials, no significant difference between control and ME/CFS was observed (*p* = 0.588), whereas for the infrequent trials, a difference in P300 amplitudes was found (*p* = 0.018), with a smaller P300 potential for the ME/CFS participants (M = 7.12, SD = 5.09) compared to the control group (M = 11.53, SD= 6.05).

**Figure 3:**
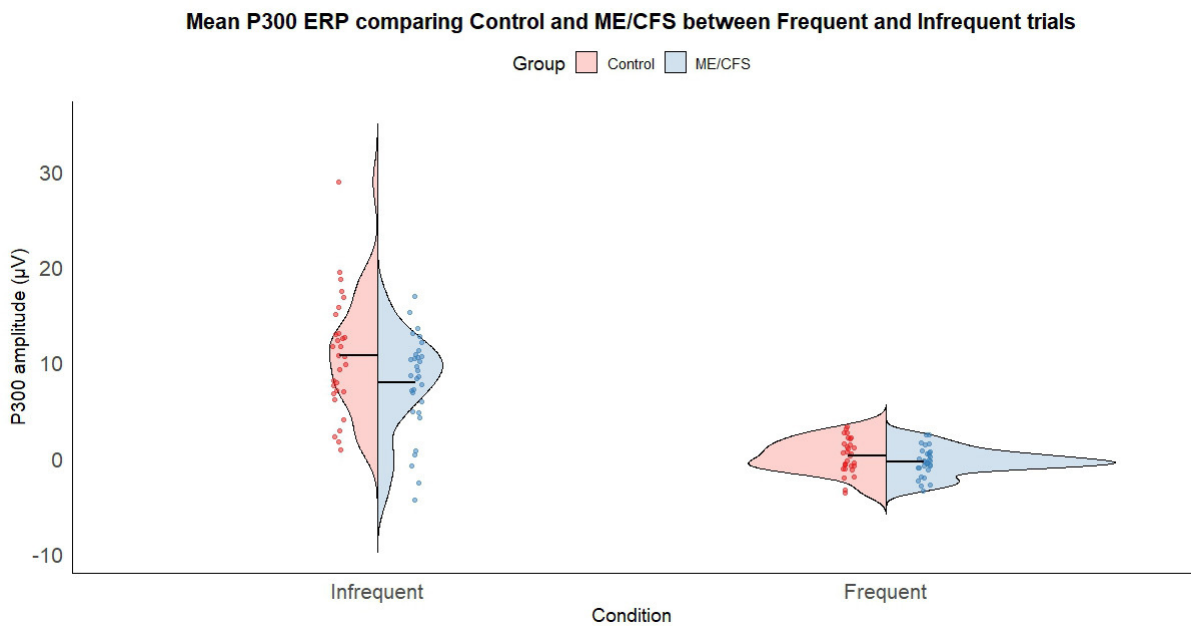
Violin chart comparing the Frequent and Infrequent oddball conditions between the control (red) and ME/CFS (blue) groups.

**Figure 4:**
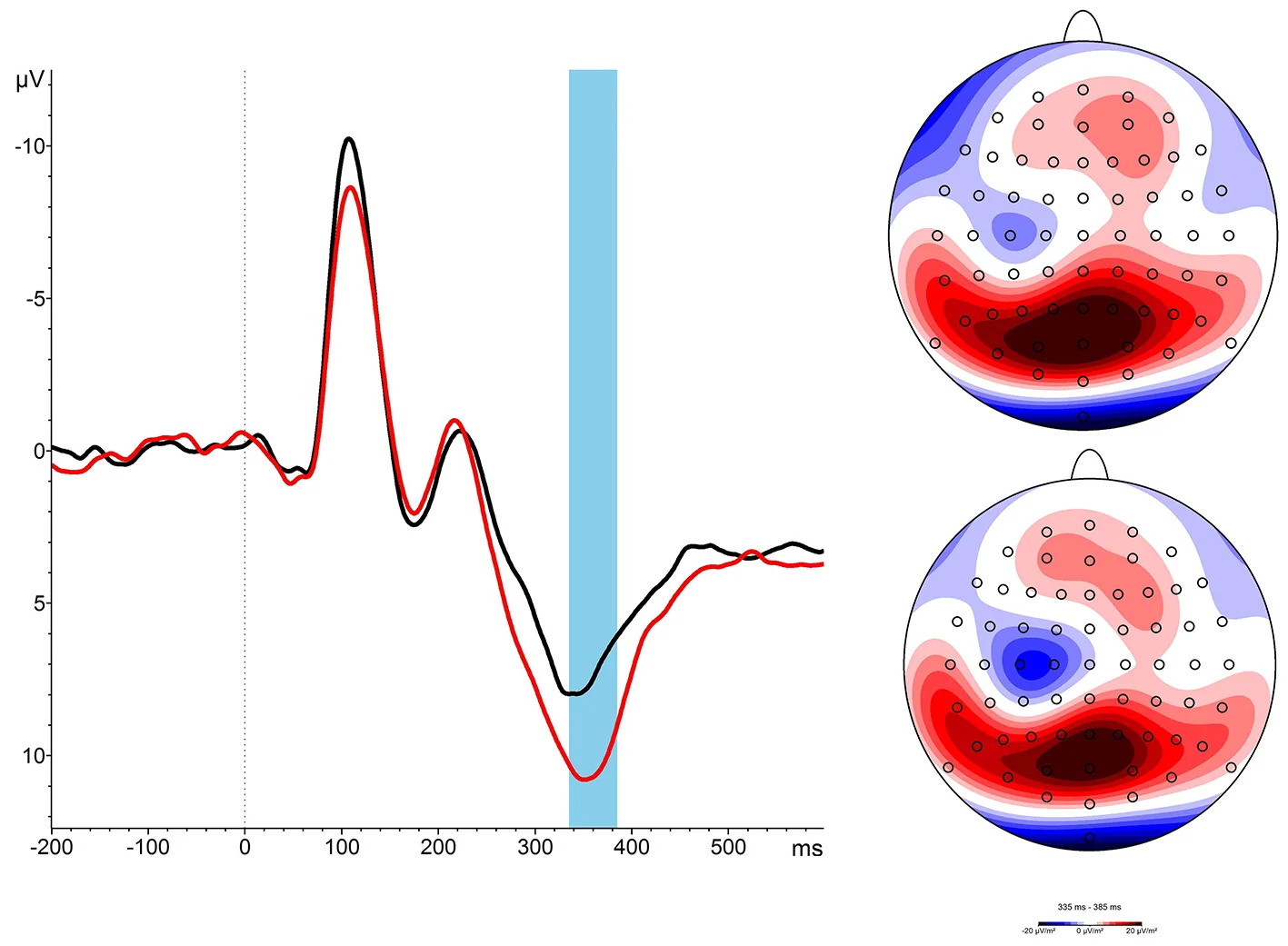
ERPs from the CPz electrode and topographical map for the P300 time-window. The Black line (bottom map) shows ERP activities for the ME/CFS-group, Red line (top map) shows for the control group.

A main effect was also observed for P300 Frequency, *F*_(1, 56)_ = 39.21, *p* < 0.001, η*p*^2^ = 0.41, indicating a large effect size with higher positive amplitude for infrequent (M = 9.29, SD = 5.72) than frequent stimuli (M = −0.056, SD = 1.65), 95% CI [−10.66, −8.10]. Plus a main effect of Group, *F*_(1, 56)_ = 5.02, *p* = 0.029, η*p*^2^ = 0.08, with a medium effect size showing a higher P300 potential for the control group (M = 5.81, SD = 5.26), compared to ME/CFS (M = 3.46, SD = 5.15), 95% CI [0.25, 4.45]. The comparison between ME/CFS and control split by frequent and infrequent trials is shown in Figure 3, and the ERP waveforms for the P300 time window in Figure 4.

#### Paired click

A clear effect of Paired-Click showed P50 attenuation of S2 (M = 1.34, SD = 2.28) vs. S1 (M = 4.33, SD = 3.23): *F*_(1, 56)_ = 7.79, *p* = 0.007, $\eta_{p}^{2}$ = 0.12; 95% CI [2.08, 3.93]. A similar pattern emerged for N100, where S2 (M = −4.03, SD = 2.93) was attenuated compared to S1 (M = −10.33, SD = 5.35): *F*_(1, 56)_ = 18.03, *p* < 0.001, $\eta_{p}^{2}$ = 0.24; 95% CI [−7.68, −4.94]. The same attenuation was observed for P200, with S2 (M = 3.84, SD = 3.23) following S1 (M = 11.67, SD = 6.29): *F*_(1, 56)_ = 11.72 *p* = 0.001, $\eta_{p}^{2}$ = 0.17; 95% CI [6.16, 9.51].

**Figure 5:**
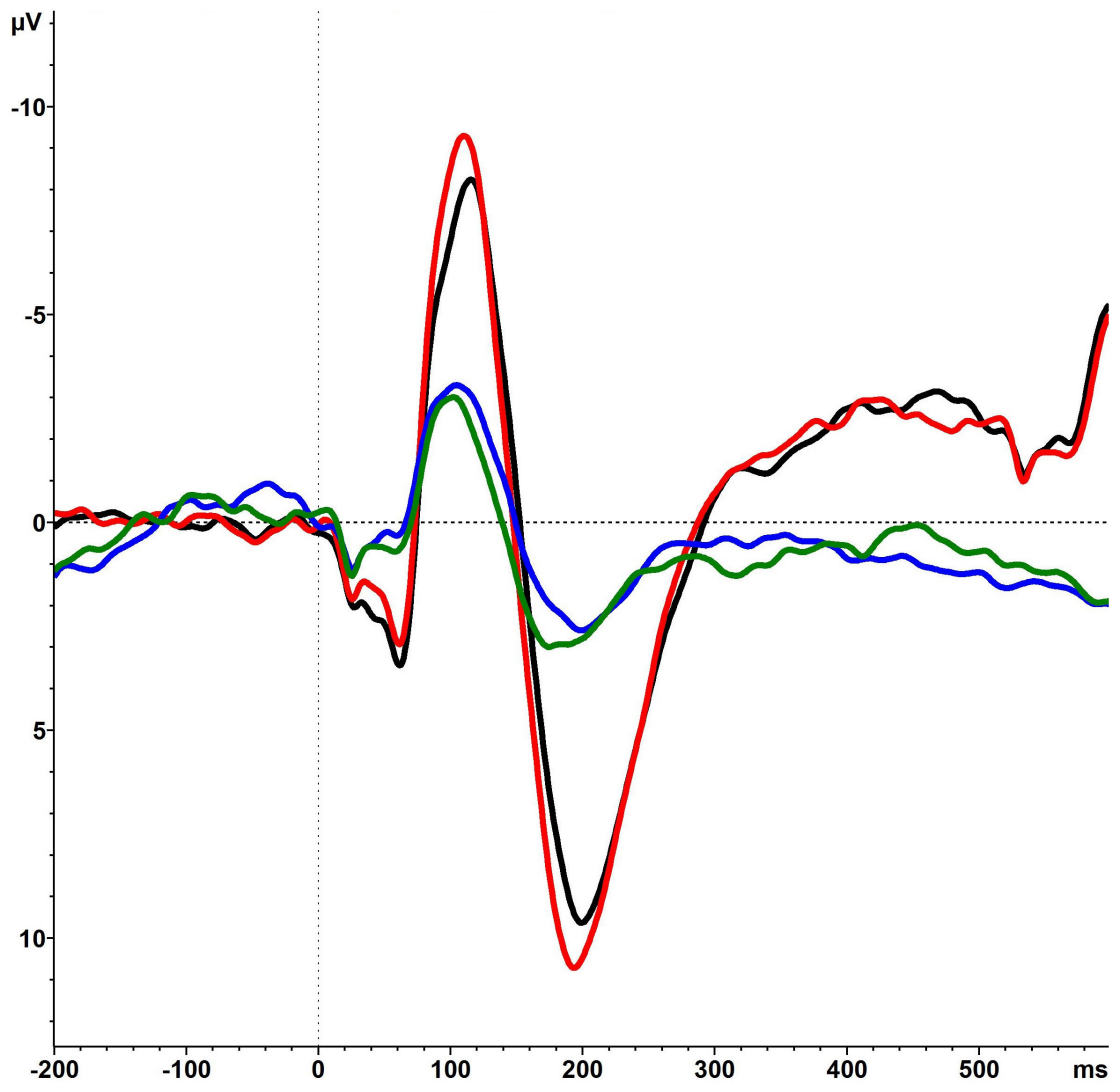
The figure shows the ERP components of the Paired-Click paradigm. Although a general attenuation of the P50, N100, and P200 is visible (Black and Red line is S1 vs. Green and Blue line of S2), similar ERPs related to auditory paired click stimuli are observed for the ME/CFS-Group (Red and Green) vs. Control (Black and Blue). S1, Stimulus 1; S2, Stimulus 2.

No main effect for Group on P50 was found, *F*_(1, 56)_ = 2.90, *p* = 0.094; N100, *F*_(1, 56)_ = 0.45, *p* = 0.505; or P200, *F*_(1, 56)_ = 0.77, *p* = 0.385. No interaction between Group and Paired-Click for P50 occurred, *F*_(1, 56)_ = 2.06, *p* = 0.156; N100, *F*_(1, 56)_ = 0.02, *p* = 0.892; or P200, *F*_(1, 56)_ = 0.47, *p* = 0.497. The ERP effects linked to S1 and S2 stimuli in the paired click task are shown in Figure 5.

### ERP and questionnaire correlations

Correlation analyses were conducted between self-reported measures and ERP indices where the two groups differed. Comparing the DSQ-SF scores for severity and frequency with the P300 EEG data on frequent and infrequent trials revealed weak negative correlations for both DSQ-S and DSQ-F in frequent [*r*(61) = −0.27, *p* = 0.039; *r*(61) = −0.27, *p* = 0.038] and infrequent trials [*r*(61) = −0.25, *p* = 0.048; *r*(61) = −0.28, *p* = 0.028]. This suggests a weak negative relation between the severity and frequency of ME/CFS symptoms on the DSQ-SF and brain potential within the P300 time window for both stimulus types.

## Discussion

ME/CFS is a neurological disorder featuring diverse neurological, cognitive, affective, physical, and physiological symptoms. Symptoms like heightened sensitivity to sensory stimuli (e.g., light, sound, touch) are noted in ME/CFS patients (8). The present study investigates the Electrophysiological correlates behind sensory issues, which are often overlooked despite their association with ME/CFS.

The ME/CFS group showed higher scores in low registration, sensory sensitivity, and sensation-avoiding self-reports, but scored lower in sensation seeking. Individuals with higher low registration need stronger stimuli to react, possibly missing some stimuli. Increased sensory sensitivity suggests a quick response to weak stimuli, highlighting greater environmental awareness and a passive response pattern. High scores in low registration and sensitivity may indicate that individuals notice details but still miss them, creating erratic and frustrating responses. Similar processing disturbances, including pupillary reflex issues, have been observed in ME/CFS (10). Wirth et al. argue that sensory hypervigilance leads to increased stimulus intake but impaired processing due to reduced cerebral blood flow, causing possible stimulus overload and hypersensitivities. The ME/CFS group's high sensation-avoiding score reflects feelings of being overwhelmed, leading to active avoidance of environmental stimuli, consistent with lower sensation-seeking scores, indicating a lack of interest in sensory experiences.

Aside from subjective measures of sensory problems, brain-based ERPs related to sensory stimuli were measured in this study. Participants performed two tasks: an auditory paired click task and an auditory oddball task, which investigated early and late stages of sensory processing, respectively. The early stage relies on stimulus characteristics, while late-stage processing is top-down, supported by higher-order brain systems. In this study, P50 suppression, as an index of early sensory gating, was examined. A clear P50 attenuation for S2 was found in both the groups (ME/CFS and Control), and similar attenuation of N100 and P200 components in the paired click task was observed for S2 in both the groups. However, the two groups did not differ in modulation for P50, N100, or P200, suggesting similar early sensory processing responses. A previous study found that 43% of their ME/CFS patients had a normal P50 S1/S2 ratio. In contrast, the rest had an abnormal ratio, with anxiety and depression as possible factors for abnormal S2 modulation (39). However, their study lacked a control group for determining whether P50 abnormality is characteristic of ME/CFS. The current study used a control group, and anxiety and depression were controlled for in the analysis. A previous large study on children with CFS found no abnormalities in the early ERP P100, N150, and P200 components (40). Likewise, other studies found no evidence of early sensory processing abnormalities in ME/CFS patients across various physiological measures (20, 41). Therefore, our findings align with those of other studies, suggesting that there are no specific early sensory processing issues in ME/CFS. However, these direct observations conflict with numerous structural and functional neuroimaging studies indicating abnormalities in the sensory brain areas (4, 5, 42, 43). Notably, these neuroimaging results on sensory brain areas were not directly linked to tasks assessing sensory processing; these studies' tasks were higher-order cognitive tasks where sensory area-related abnormalities were observed.

The ME/CFS patients in our study showed significant sensory processing issues. However, we found no evidence of early sensory processing problems at the electrophysiological level, as indexed by ERP components in the auditory paired-click paradigm. We further investigated higher-order brain processes in ME/CFS patients using the oddball task, focusing on the P300 component. Previous studies have indicated P300 ERP component latency or amplitude abnormalities in ME/CFS patients. For example, Prasher et al. (20) found that patients with abnormal P300 components had associated memory and attention problems compared to those without abnormalities. However, other studies have not found evidence of late-stage problems in information processing. An ERP study compared pre- and post-stimulus processing in ME/CFS patients vs. a healthy group (41). Their findings showed ME/CFS patients had issues in pre-stimulus motor-related processes, but no problems with post-stimulus sensory (N100) or cognitive (P300) brain processes. Similarly, Polich et al. (21) did not find differences in post-sensory (N100) or cognitive (P300) ERP components between ME/CFS and healthy controls. However, the present study found an overall reduction in P300 potential in ME/CFS patients compared to healthy controls. In short, the Oddball response was reduced in participants with ME/CFS compared to controls.

P300 reflects as markers for inhibitory brain processes, indexing updates in working memory and cognitive task completion (44). They are also influenced by unexpected stimuli, reflecting an evaluative top-down process to identify discrepancies between task context and sensory input (45). Palidis et al. (46) found that P300 potentials increase with the learning rate of visual sensory changes. Reduced P300 potential in the ME/CFS patients suggests a failure to manage sensory changes and evaluate sensory stimulus relevance. We propose that sensory processing issues in ME/CFS may be indicative of an altered cognitive and attentional top-down mechanisms for evaluating, learning from, and updating working memory in changing sensory environments. Similar issues have been noted in other conditions linked to higher-order brain processes. For example, Micoulaud-Franchi et al. (47) reported sensory gating problems in attention deficit hyperactivity disorder patients, associated with diminished higher-order processes indexed by P300. As P300 potentials could also be affected by several other factors such as generalized fatigue, a feature of ME/CFS, and/or motivation, a systematic investigation is needed to isolate the specific roles of these factors in P300 potential changes in ME/CFS.

This novel study systematically examined the electrophysiological mechanisms involved in higher-order and early sensory processing in ME/CFS patients. This systematic evaluation of sensory processing issues in ME/CFS has not been previously reported. Based on our systematic investigation, we propose that the brain systems associated with higher-order processing may be contributing to sensory processing problems in ME/CFS. Such findings have significant therapeutic implications. We suggest that any intervention addressing sensory issues in ME/CFS patients should focus on strategies targeting top-down brain modulatory processes, rather than the early sensory processing system.

The findings are novel and informative but should be viewed considering potential limitations. The experiment was lab-based, testing only one sensory modality, whereas real-world sensory information comes from multiple modalities. Additionally, although we had a modest sample size and medical specialists diagnosed all ME/CFS patients, their illness durations varied significantly. We acknowledge that we may not have recruited patients with higher symptom severity. Furthermore, the observed effect sizes for the P300 difference between the patient and control groups are smaller which warrants cautious interpretations of our findings. Although we statistically controlled for the gender differences, the influence of gender differences on P300 potential differences between the patient and control groups cannot be fully ruled out. P300 potentials are known to be affected by gender differences and the findings should be interpreted with caution. Nonetheless, the study's findings provide valuable insights into sensory processing issues in ME/CFS, likely informing interventions for sensory problems in these patients.

In conclusion, the ME/CFS group showed reduced amplitude in the P300 time window for the infrequent trials compared to controls, indicating suboptimal brain responses for working memory and response inhibition in these patients.

## Data Availability

The raw data supporting the conclusions of this article will be made available by the authors, without undue reservation.
