# Correction: Higher-order brain processes, rather than early processing, underlie sensory problems in ME/CFS: evidence from ERPs

**DOI:** 10.3389/fmed.2026.1917331

**Published:** 2026-08-19

**Authors:** Sanjay Kumar, Alfred Veldhuis, Farzaneh Yazdani

**Affiliations:** 1 Department of Psychology, Social Work and Public Health, Oxford Brookes University, Oxford, United Kingdom; 2 School of Life Sciences, University of Roehampton, London, United Kingdom; 3 Occupational Therapy, Oxford Brookes University, Oxford, United Kingdom

**Keywords:** brain, chronic sensation, cognition, electroencephalography, fatigue syndrome

The captions for Figures 1 and 2 were incorrectly published as:

Figure 1. Mean scores on the Sensory Profile questionnaire for the ME/CFS and Control groups. The error bar represents 1 standard error of the mean. All comparisons between ME/CFS and Control are significant.

Figure 2. Schematic representation of Experiment 1: Oddball (top) and Experiment 2: Paired-Click (bottom). Experiment 1 had low pitch (600 Hz) and high pitch (1,600 Hz), frequent and infrequent stimuli. The high-pitched stimuli occurred randomly in the sequence. For the Paired-Click, all stimuli were 2,000 Hz, and S1 and S2 were always 500 ms apart.

The correct captions are:

Figure 1. Schematic representation of Experiment 1: Oddball **(Top)** and Experiment 2: Paired-Click **(Bottom)**. Experiment 1 had low pitch (600 Hz) and high pitch (1,600 Hz), frequent and infrequent stimuli. The high-pitched stimuli occurred randomly in the sequence. For the Paired-Click, all stimuli were 2,000 Hz, and S1 and S2 were always 500 ms apart.

Figure 2. Mean scores on the Sensory Profile questionnaire for the ME/CFS and Control groups. The error bar represents 1 standard error of the mean. All comparisons between ME/CFS and Control are significant.

The figures themselves are correctly placed; only the captions were transposed.

The original version of this article has been updated.

